# Importance of Microvascular Invasion Risk and Tumor Size on Recurrence and Survival of Hepatocellular Carcinoma After Anatomical Resection and Non-anatomical Resection

**DOI:** 10.3389/fonc.2021.621622

**Published:** 2021-03-17

**Authors:** Haoyu Hu, Shuo Qi, Silue Zeng, Peng Zhang, Linyun He, Sai Wen, Ning Zeng, Jian Yang, Weiqi Zhang, Wen Zhu, Nan Xiang, Chihua Fang

**Affiliations:** ^1^Department of Hepatobiliary Surgery, Zhujiang Hospital, Southern Medical University, Guangzhou, China; ^2^Guangdong Provincial Clinical and Engineering Center of Digital Medicine, Guangzhou, China

**Keywords:** hepatocellular carcinoma, microvascular invasion, prediction model, tumor size, hepatectomy

## Abstract

**Purpose:** To establish a valid prediction model to prognose the occurrence of microvascular invasion (MVI), and to compare the efficacy of anatomic resection (AR) or non-anatomic resection (NAR) for hepatocellular carcinoma (HCC).

**Methods:** Two hundred twenty-eight patients with HCC who underwent surgical treatment were enrolled. Their hematological indicators, MRI imaging features, and outcome data were acquired.

**Result:** In the multivariable analysis, alpha-fetoprotein >15 ng/mL, neutrophil to lymphocyte ratio >3.8, corona enhancement, and peritumoral hypointensity on hepatobiliary phase were associated with MVI. According on these factors, the AUROC of the predictive model in the primary and validation cohorts was 0.884 (95% CI: 0.829, 0.938) and 0.899 (95% CI: 0.821, 0.967), respectively. Patients with high risk of MVI or those with low risk of MVI but tumor size >5 cm in the AR group were associated with a lower rate of recurrence and death than patients in the NAR group; however, when patients are in the state of low-risk MVI with tumor size >5 cm, there is no difference in the rate of recurrence and death between AR and NAR.

**Conclusion:** Our predictive model for HCC with MVI is convenient and accurate. Patients with high-risk of MVI or low-risk of MVI but tumor size >5 cm executing AR is of great necessity.

## Key Points

- We have developed a convenient and accurate predictive-model by combining hematological indicators with imaging features.
- It can assist surgeons choose the optimized surgical approach.
- Then it would eventually help to improve the recurrence-free and long-term survival rate of patients.

## Introduction

Vascular invasion of hepatocellular carcinoma (HCC) include macrovascular invasion and microvascular invasion in pathology, both of which are predictors of poor prognosis after surgical resection or liver transplantation (1, 2). The 5-year recurrence rate of HCC patients with microvascular invasion after radical hepatic resection is reportedly as high as 70%, and tumor recurrence rate exceeds 35% even after liver transplantation (3, 4).

Though preoperative radiological techniques such as CT and MRI are feasible to detect macrovascular invasion (5), recording the presence of microvascular invasion (MVI) is still challenging since it requires histopathological examination of surgically resected specimen (6). Early prediction of MVI in hepatocellular carcinoma remains elusive. Studies have shown that microvascular violation of state can be reflected by specific clinical hematological indicators, such as des-gamma-carboxyprothrombin (PIVKA-II), alpha-fetoprotein (AFP), and peripheral neutrophil to lymphocyte ratio (NLR) (7, 8); also, it can be predicted by tumor size, multiple tumor nodules, tumor rough edges, incomplete capsule, and nuclear magnetic resonance arterial peritumoral enhancement imaging characteristics and changes in peritumoral hepatobiliary specific density of microvessels (9–12). Currently, Radiomics is the most popular method for microvascular invasion assessment and prediction (13). Despite the potential of Radiomics to guide clinical decision making, there is a lack of standardized evaluation toward numerous published Radiomics studies; moreover, Radiomics necessitates interdisciplinary cooperation. These two factors are the reason why Radiomics is difficult to be implemented in many hospitals. Therefore, a simple and effective method capable of predicting the incidence of MVI prior to surgery is urgently needed to improve prognosis after radical resection in patients with HCC.

Additionally, it has been reported that anatomical resection (AR) of the liver can be useful in isolating microvascular metastases while removing lesions (14, 15). However, multicenter retrospective studies revealed no significant difference between AR and non-anatomical resection (NAR) in terms of tumor-free survival and long-term survival post-operatively (16). In the current study, we aim to establish a valid prediction model to prognose the occurrence of microvascular invasion, and apply this model to compare the efficacy of AR or NAR in the treatment of patients retrospectively.

## Patients and Methods

In this study, 228 patients with HCC who underwent surgical treatment at Zhujiang Hospital, Southern Medical University, from January 2012 to June 2018 were enrolled by the following criteria. Inclusion criteria: (1) pathological diagnosis of HCC; (2) hematological indicators processed within 15 days before surgery, including complete blood count, alpha-fetoprotein (AFP) and imaging examinations; (3) Child-Pugh classification of liver function as A; (4) surgery performed under the guidance of 3D reconstructed images; exclusion criteria: (1) palliative tumor resection; (2) primary angiographic diagnosis of cancerous thrombosis; (3) patients who received preoperative radiotherapy, chemotherapy, TACE, or targeted therapy as the initial treatment such as sorafenib, anti-PD-1/PDL-1; (4) follow-up time <12 months. Finally, a total of 228 patients were included in the study. The patients were divided into two independent cohorts at a ratio of 7:3 using a random number table. One hundred sixty patients constituted the training cohort and the remaining 68 formed the validation cohort. Ethics committee approval was approved by the Institutional Ethics Review Board of Zhujiang Hospital, Southern Medical University (ethics number:2018-GDYK-001), and clinical data of the above patients were collected and analyzed retrospectively.

### Data Acquisition

1. Hematological indicators: complete blood count, liver function, AFP, HBV, and HCV antigen/antibody, and HBV deoxyribonucleic acid (HBV-DNA).
2. Imaging examination: MRI was performed with the patient relaxed in a supine position. The positioning image adopts breath-hold fast spoiled gradient echo sequences and fast imaging employing steady-state acquisition (FIESTA) on the coronal plane. The magnetic resonance spectrum (MRS) scan uses a single-element spot-resolved spectrum sequence, and the scanning time is about 1 min. For gadoxetic acid (Primovist or Eovist; Bayer Schering Pharma, Berlin, Germany)-enhanced MRI, the following images were obtained using a fat-suppressed 3-dimensional gradient-echo T1-weighted sequence (volumetric interpolated breath-hold examination, Siemens or T1 high-resolution isotropic volume examination, Philips): arterial phase (20–35 s), portal phase (60 s), delayed phase (3 min), and hepatobiliary phase (HBP) (20 min). The scanning delay time for arterial phase imaging was determined using MR fluoroscopic monitoring.

### Analysis of Hematological Indicators

NLR is the ratio of neutrophil count to lymphocyte count. The obtained hematological index is established with the ROC curve of pathological MVI information. If the area under the curve (AUC) ≧0.6, the cutoff value corresponding to the Youden index is obtained, and the variables are classified into two categories; if the AUC <0.6, the classification criteria for each hematological index are determined through the literature report.

### Analysis of Imaging Data

Preoperative MR images were retrospectively evaluated using a Picture Archiving and Communication System (PACS; Pathspeed, GE Medical Systems Integrated Imaging Solutions, Mt. Prospect, IL, USA). Image analysis was performed by two abdominal radiologists (Li Xinming and Lin Huan, with 7 and 6 years of experience in hepatic MRI, respectively) who were unaware of information on clinical, laboratory, pathologic, and follow-up results. The two reviewers evaluated the following imaging features for each HCC independently: (a) arterial rim enhancement (17), means in the arterial phase existing irregularity like enhancement with relatively hypovascular central areas; (b) corona enhancement (C E) (18), known as the transient enhancement of the perilesional parenchyma. The enhancement of the perilesional parenchyma fades in the portal venous phase and is resolved by the 3-min delayed phase; (c) radiological capsule (18), defined as a peripheral rim of smooth hyperenhancement in the portal venous or delayed phase; (d) tumor margin (19), categorized as non-smooth margin, showing as non-nodular tumors with an out-of-shape margin that had the edge-peaked distribution, or smooth margin, showing as nodular tumors with smooth outline in the HBP images; (e) tumor hypointensity on HBP (20), means comparing with the surrounding liver, the tumor shows lower signal intensity on HBP; (f) peritumoral hypointensity on HBP(HY-HBP) (21), defined as wedge-shaped or flame-like hypointense area of hepatic parenchyma located outside of the tumor margin on HBP.

### Surgical Planning and Procedure

**Figure 1:**
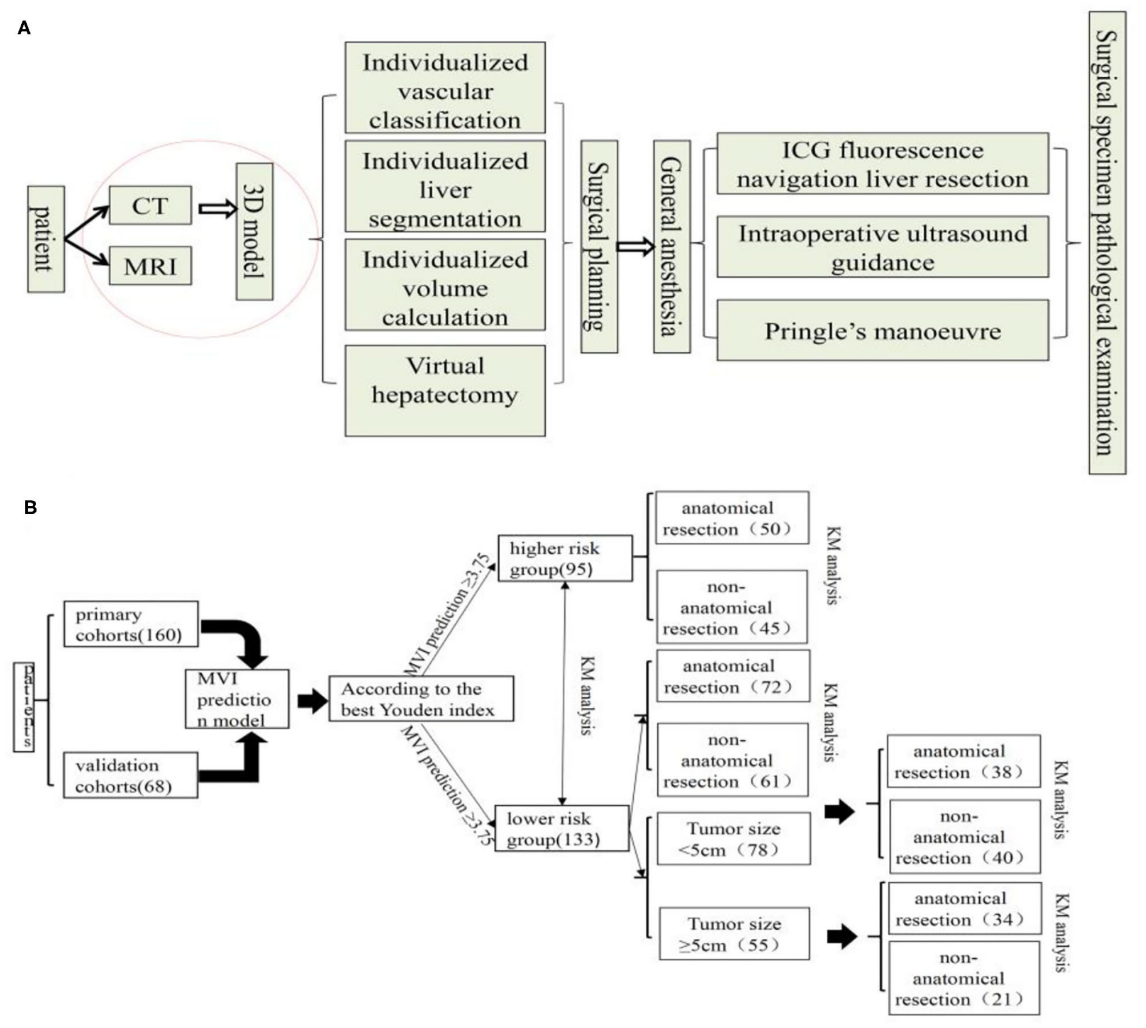
Surgical planning and procedure **(A)** and flow chart of manuscript design **(B)**, KM, Kaplan-Meier.

All patients completed a three-dimensional visualization analysis before surgery (22). The procedure of surgical planning is shown in Figure 1A. Most resections were intended to be anatomic according to the vascular topological relationship. However, in a few patients with peripheral lesions and portal hypertension or suboptimal liver function, partial resection including the tumor and an intended 1–2 cm margin were performed.

### Pathological Analysis

All surgical specimens were examined by two pathologists to detect the presence of MVI. The histologic parameters ordinarily included pathological grade, size, number, surgical margin, and MVI status of the resected tumor were based on the practice guidelines for the pathological diagnosis of primary liver cancer: 2015 update (23). MVI was defined as the presence of a tumor in a microportal vein, microhepatic vein, or a capsular vessel of the surrounding liver tissue lined by the endothelium that was visible only on microscopy.

### Follow-Up

Follow-up examinations were conducted 1 month after surgery and then every 2–3 months using laboratory findings (complete blood count, serum AFP, and liver function). Abdominal ultrasonography and contrast-enhanced CT or MR were performed every 3 months. The patients were followed-up once every 3–4 months post-operatively until death or dropout from the follow-up program. A diagnosis of recurrence of HCC was based on CT and/or MRI and elevated serum a-fetoprotein (AFP) levels. Most of the patients were observed according to the recommendation guidelines for diagnosis and management of liver diseases by the Chinese “Guidelines for the Diagnosis and Treatment of Primary Hepatocellular Carcinoma (2017 edition).”

### Statistical Analysis

Univariate logistic regression analyses were performed to determine factors with MVI risk; multivariate analyses with an adjusted odds-ratio (OR) regression model were conducted to construct the MVI risk model from multi-scale hematological indicators and Radiomic signatures. The candidate clinical variables were sex, age, history of hepatic virus infection (0, negative; 1, history of HBV, HBV + HCVB), history of cirrhosis (0, absent; 1, present), AST (0, ≦34 U/L; 1, >34 U/L), ALT (0, ≦40 U/L; 1, >40 U/L), PT (0, ≦13 s, 1, >13 s), TBil (0, ≥35 g/L, 1 <35 g/L), AFP (0, ≦15 ng/mL; 1, >15 ng/mL), NLR (0, ≦3.8; 1, >3.8), Tumor size (0, ≤ 5 cm; 1 >5 cm). Radiologic features included arterial rim enhancement, arterial peritumoral enhancement, tumor margin, radiological capsule, tumor hypointensity on HBP, and peritumoral hypointensity on HBP. In order to partition the patients into high- and low-risk MVI groups, the optimal cutoff value for the risk scores was determined via area under the ROC curve (AUROC) analysis using the Youden index.

The rate of HCC recurrence and survival between the AR and NAR groups based on MVI risk in the prediction model was subsequently compared. In subgroup analysis, this rate in the two groups was assessed based on high risk of MVI based on the prediction model. Also, In the MVI low-risk group, the tumor diameter of 5 cm was used as the cut-off point to compare the survival difference between AR and NAR flow of manuscript design showed in Figure 1B. All statistical analyses were performed using SPSS version 25.0 (SPSS Inc., Chicago, IL, USA). The level of statistical significance was set at *P* < 0.05.

## Results

### Patient Characteristics

The baseline data of the patients are shown in Table 1 as the primary cohort and validation cohort. Among the two cohorts, male patients predominate over females. There were no differences between the two cohorts. Also, the size of the tumor in the primary cohort is 5.44 ± 3.17 cm and 4.14 ± 2.26 cm in the validation cohort, which shows no significant difference (*P* = 0.076). Histopathology shows that the number of MVI cases in the two groups is 56 and 20, respectively, with no significant difference.

**Table 1:** Baseline characteristics of study patients.

**Variable**	**Primary cohort**	**Validation cohort**	***P*-value**
Age(Mean ± SD, years)	50.74 ± 11.58	51.25 ± 12.08	0.766
M/F ratio	129/31	51/17	0.341
History of hepatic virus infection			0.148
Yes/No	128/32	41/17	
Liver cirrhosis			0.549
Presence/Absence	76/84	35/33	
AFP level (median, Q, ng/mL)	24.50 (4.15, 332.00)	4.710 (2.388, 54.69)	0.059
NLR level (median, Q)	2.46 (1.64, 5.74)	2.92 (1.93, 4.98)	0.259
ALT (Mean ± SD, U/L)	43.42 ± 37.88	39.43 ± 38.79	0.471
AST (median, Q, U/L)	35 (24, 45)	27 (21, 47)	0.122
TBil (median, Q, μmol/L)	13.77 ± 3.53	16.87 ± 1.98	0.911
ALB (Mean ± SD, g/L)	39.50 ± 5.59	38.44 ± 1.91	0.141
PT (Mean ± SD,^*^10^9^)	13.65 ± 1.51	13.78 ± 1.04	0.533
Intraoperative blood loss, (median, Q, mL)	400 (200, 600)	300 (200, 575)	0.255
Anatomical resection			>0.999
Yes/Not	86/74	36/32	
Anatomical lobectomy			0.812
Segment	10	6	
1 lobe	30	12	
2 lobe	45	18	
≥3 lobe	1	0	
Tumor size (Mean ± SD, cm)	5.44 ± 3.17	4.14 ± 2.26	0.076
Satellite nodules	20	11	0.6836
Tumor number			0.603
1/2/3	140/18/2	57/9/2	
Capsule formation			0.149
Presence/Absence	70/90	37/31	
Pathological grade			0.611
Well differentiated	26	8	
Moderately differentiated	111	48	
Poorly differentiated	23	12	
MVI			0.446
Presence/Absence	56/104	20/48	
Arterial rim enhancement			0.197
Presence/Absence	76/84	37/31	
corona enhancement			0.317
Presence/Absence	81/79	37/31	
Tumor margin(rough)			0.229
Presence/Absence	95/65	31/37	
Radiological capsule			0.883
Presence/Absence	90/70	40/28	
Tumor hypointensity on HBP			>0.999
Presence/Absence	141/19	60/8	
Peritumoral hypointensity on HBP			0.148
Presence/Absence	84/76	28/40	

*AFP, alpha fetoprotein; NLR, neutrophil to lymphocyte ratio; ALT, alanine aminotransferase; AST, aspartate aminotransferase; PT, prothrombin time; TBil, total bilirubin; ALB, albumin; MVI, microvascular invasion*.

**Figure 2:**
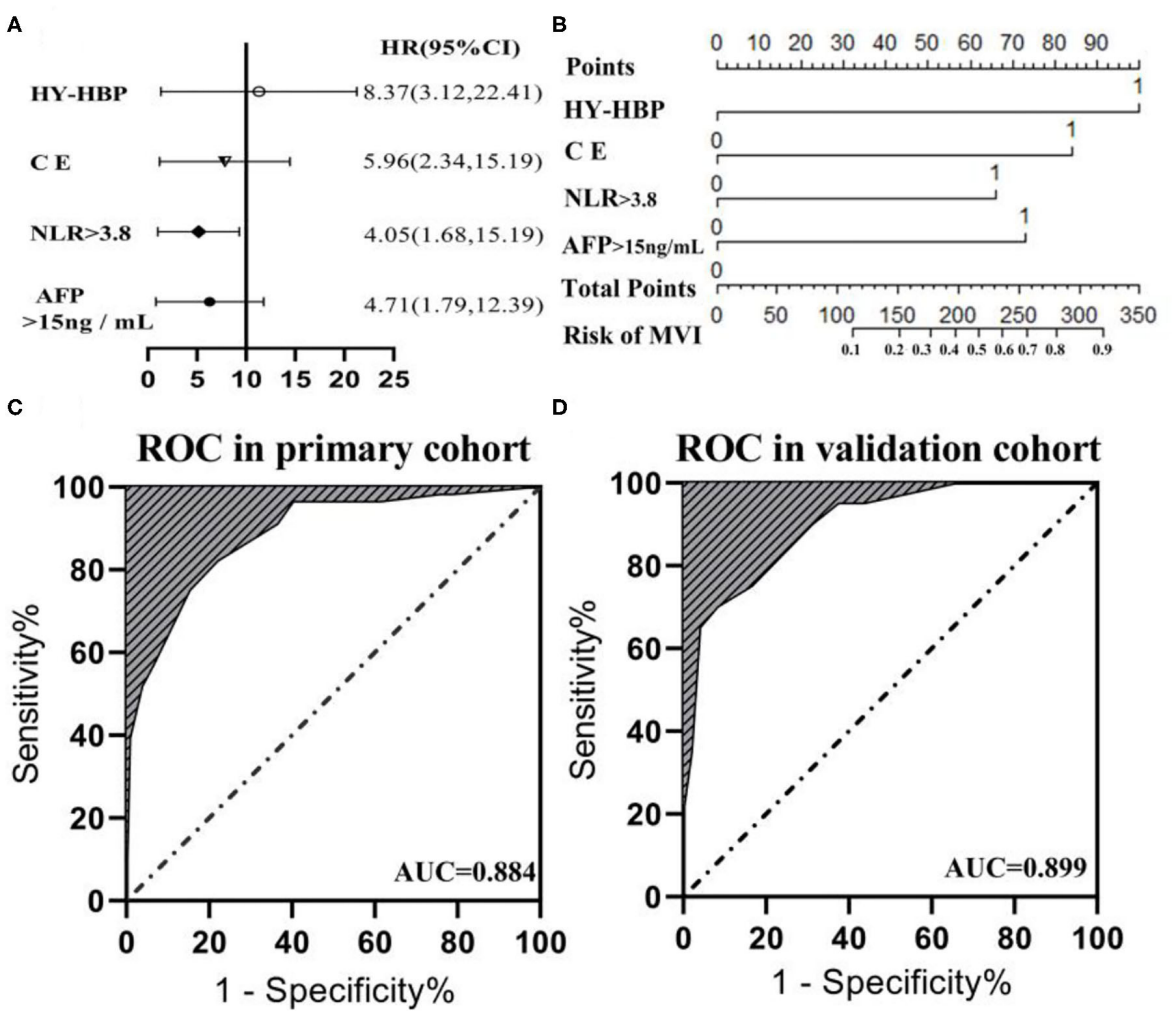
**(A)** Forest plot of independent predictors of MVI with odds-ratio (OR) multivariate regression model. **(B)** The model presented with a nomogram scaled by the proportional regression coefficient of each risk variables. The area under the receiver operating characteristic curve (AUROC) for the MVI prediction model: **(C)** AUROC was 0.884 in the primary cohorts; **(D)** AUROC was 0.899 in the validation cohorts.

The six risk factors related to MVI were screened by single factor logistic regression analysis using laboratory hematology examination indicators and typical imaging characteristics, respectively: AFP >15 ng/mL (OR: 5.647, *P* < 0.001), NLR >3.8 (OR: 7.970, *P* < 0.001), AST >34 U/L (OR: 2.724, *P* = 0.003), Arterial rim enhancement (OR: 0.492, *P* = 0.03), corona enhancement (C E, OR: 6.319; *P* < 0.001), peritumoral hypointensity on HBP (PH-HBP, OR: 7.510; *P* < 0.001), as shown in Table 2; and AFP >15 ng/mL (OR: 5.411; 95% CI: 2.093, 13.990; *P* < 0.001), NLR >3.8 (OR: 3.977; 95% CI: 1.689, 9.368; *P* = 0.002), C E (OR: 6.183; 95% CI: 2.478, 15.429; *P* < 0.001), PH-HBP (OR: 8.754; 95% CI: 3.355, 22.843; *P* < 0.001; Table 3); the MVI prediction model is: MVI risk = 1.5 × AFP + 1 × NLR + 2 × C E + 2 × PH-HBP, obtained by adding the total number of points scored in each of the four independent risk factors. The highest score is 6.5, and the lowest score is 0. Through the multi-factor logistic regression analysis in Table 3. A forest plot of independent predictors of MVI with odds-ratio and a nomogram plot for predicting MVI risk (Figure 2A) and the above four factors was constructed (Figure 2B). In the primary cohort, the AUROC (Figure 2C) of the nomogram was 0.887 (95% CI: 0.835, 0.939). In order to distinguish the MVI high-risk group and the low-risk group from the whole sample, we obtained an optimal cutoff value of 3.75.

**Table 2:** Univariable and multivariable analysis of preoperative data for presence of microvascular invasion in the primary cohort.

**Variable**	**Univariable analysis**		**Multivariable analysis**	
	**OR (95% CI)**	***P*-value**	**OR (95% CI)**	***P*-value**
History of hepatic virus infection	0.83 (0.39, 1.75)	0.625		
Liver cirrhosis	0.85 (0.54, 1.35)	0.494		
AFP >15 ng/mL	5.15 (2.40, 11.08)	<0.001	4.30 (1.61, 11.48)	0.002
NLR >3.8	7.44 (3.60, 15.39)	<0.001	4.21 (1.70, 10.39)	0.002
Platelet count >100 (10^9^/L)	1.86 (0.58, 5.99)	0.300		
ALT	1.09 (0.55, 2.13)	0.809		
AST	2.10 (1.07, 4.13)	0.031	1.31 (0.52, 3.32)	0.567
TBil	0.75 (0.25, 2.25)	0.610		
ALB	0.90 (0.47, 1.73)	0.753		
PT	0.87 (0.44, 1.73)	0.70		
Tumor size	1.30 (0.68, 2.50)	0.426		
Arterial rim enhancement	0.46 (0.24, 0.90)	0.02	0.62 (0.25, 1.59)	0.321
Corona enhancement	6.64 (3.12, 14.11)	0.001	5.40 (2.04, 14.30)	<0.001
Tumor margin	1.37 (0.70, 2.69)	0.354		
Radiological capsule	1.06 (0.55, 2.04)	0.870		
Tumor hypointensity on HBP	1.40 (0.51, 3.85)	0.509		
Peritumoral hypointensity on HBP	7.99 (3.62, 17.64)	<0.001	8.06 (2.96, 21.93)	<0.001

*HBP, hepatobiliary phase*.

**Table 3:** Multivariable analysis of risk factors of MVI and measurement of the MVI risk score.

**Variable**	**Multivariable analysis**			
	**OR (95% CI)**	***P*-value**	**B coefficient**	**Points**
AFP >15 ng/mL	4.71 (1.79, 12.39)	0.002	1.55	1.5
NLR >3.8	4.05 (1.68, 9.75)	0.002	1.40	1
corona enhancement	5.96 (2.34, 15.19)	<0.001	1.78	2
Peritumoral hypointensity on HBP	8.37 (3.12, 22.41)	<0.001	2.12	2

*Multivariable logistic regression model was carried out by using stepwise backward variable selection. The scaled coefficients were simplified by rounding them to nearest half. The MVI risk score is obtained by adding the total number of points scored in each of the 4 variables*.

### Model Validation

**Figure 3:**
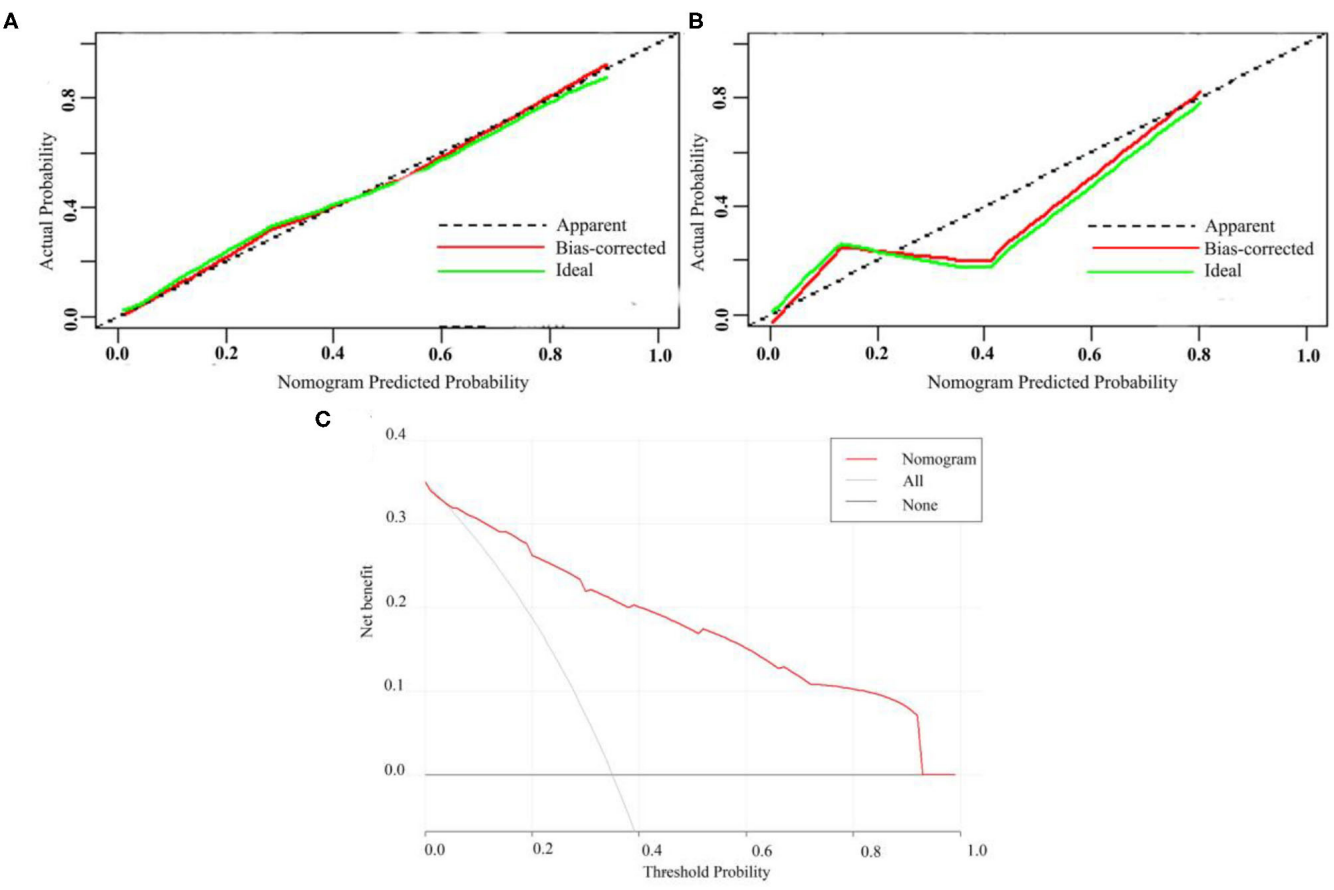
Calibration curve of MVI prediction model in primary cohort **(A)** and validation cohort **(B)**. **(C)** Presents the decision curve for all patients.

A calibration analysis of the MVI prediction model showed high coherence between the observed risk and the predicted risk (*P* = 0.200) in the primary cohort (Figure 3A) and validation cohort (Figure 3B); meanwhile, the AUROC of the model in the validation cohort (Figure 2D) was 0.899 (95% CI: 0.821, 0.967), with the sensitivity of 0.750, the specificity of 0.833, and accuracy of 0.794.while in the primary cohort the average AUROC was 0.884 (95% CI: 0.829, 0.938) and its sensitivity, specificity, and accuracy were 0.824, 0.779, and 0.795, respectively.

Figure 3C presents the decision curve. It shows that if the threshold probability is within a range from 0.01 to 0.92, the use of nomogram model can bring more net benefit than the patient of complete intervention or no intervention at all.

### Recurrence and Survival

**Figure 4:**
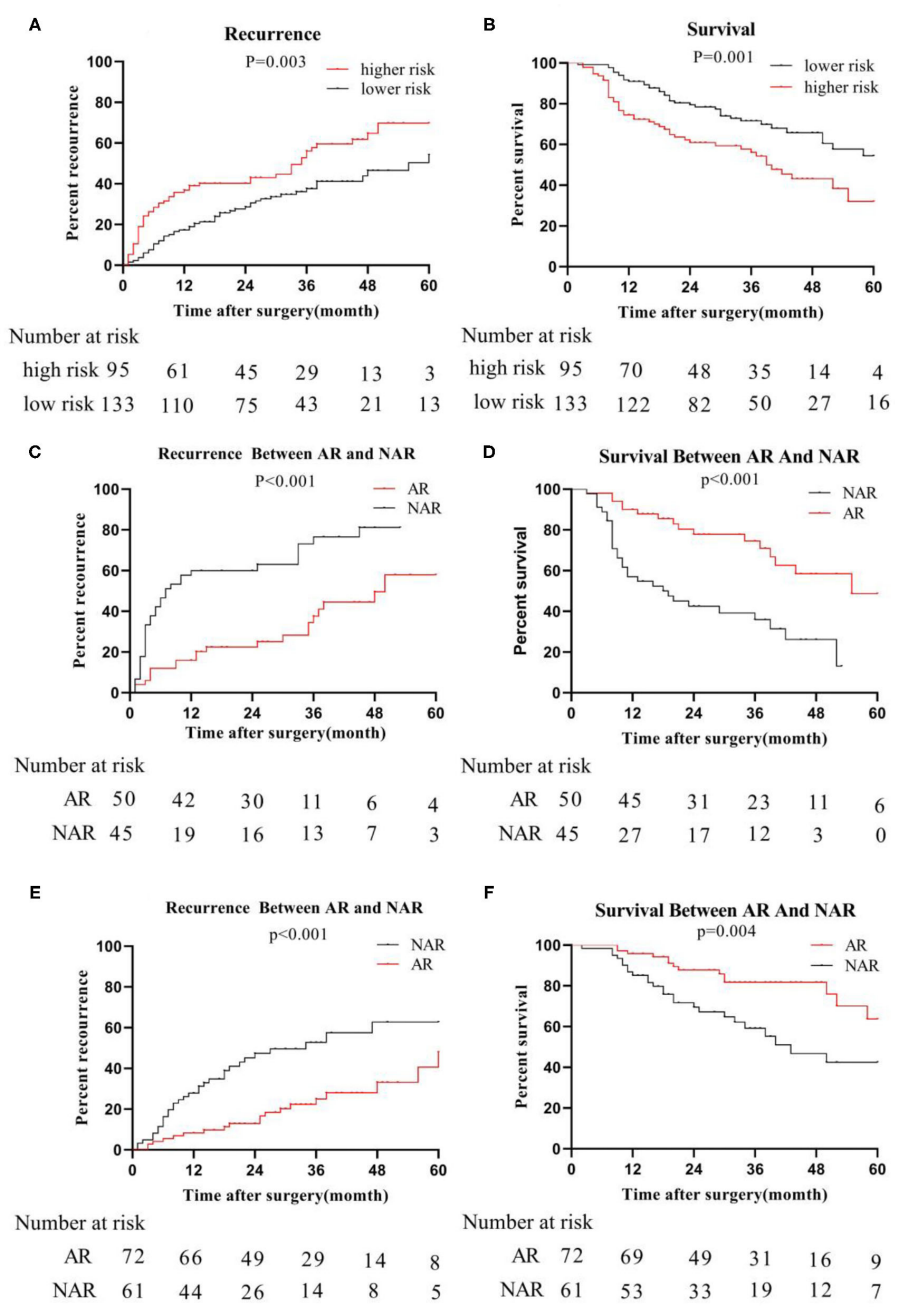
Recurrence and survival of the whole cohort; we organize the primary cohort and the validation cohort into one cohort, recurrence-rate **(A)** and survival-rate **(B)** comparison between higher risk group and lower risk group; in higher risk group, recurrence-rate **(C)** survival -rate **(D)** comparison between AR and NAR; in lower risk group, recurrence-rate **(E)** and survival -rate **(F)** comparison between AR and NAR.

The primary cohort and the validation cohort were organized into one cohort, and then divided into a MVI high-risk group and a MVI low-risk group by the MVI prediction model. The median recurrence time in the high-risk group was 18 months, and 28 months in the low-risk group. The difference was statistically significant (*P* = 0.003; Figure 4A). The 3- and 5-year survival rates of the higher-risk group were 56.09 and 71.59%, respectively, which were significantly lower than the 32.01% and 54.47% of the lower risk group (*p* = 0.001; Figure 4B). The 5-year overall recurrence rate of the AR in the high-risk group was 58.00% lower than the 5-year recurrence rate (81.20%) of the NAR (Figure 4C). The 3- and 5-year survival rates were significantly better in the group than in the NAR group. Similarly, in the MVI low-risk group, AR was higher than NAR in terms of 3- and 5-year recurrence rate, and survival rate (Figures 4D–F). Meanwhile, we obtained similar results of recurrence and survival to those obtained for real MVI treated with AR or NAR (Supplementary Figure 1).

In the high-risk group, tumor size was further considered as a risk factor affecting tumor recurrence and long-term survival in patients undergoing anatomical resection (AR)/non-anatomical resection (NAR). When the tumor size was ≦5 cm, the recurrence (*P* = 0.039) rate of the AR group was significantly lower than that of the NAR group (Supplementary Figure 2A), while the survival (*P* = 0.011) rate of the AR group was significantly higher than that of the NAR group (Supplementary Figure 2B). Similarly, when the tumor size reached >5 cm, the recurrence rate and survival rate of the AR group and NAR group were the same as when the tumor size was ≤ 5 cm (Supplementary Figures 2D,E).

**Figure 5:**
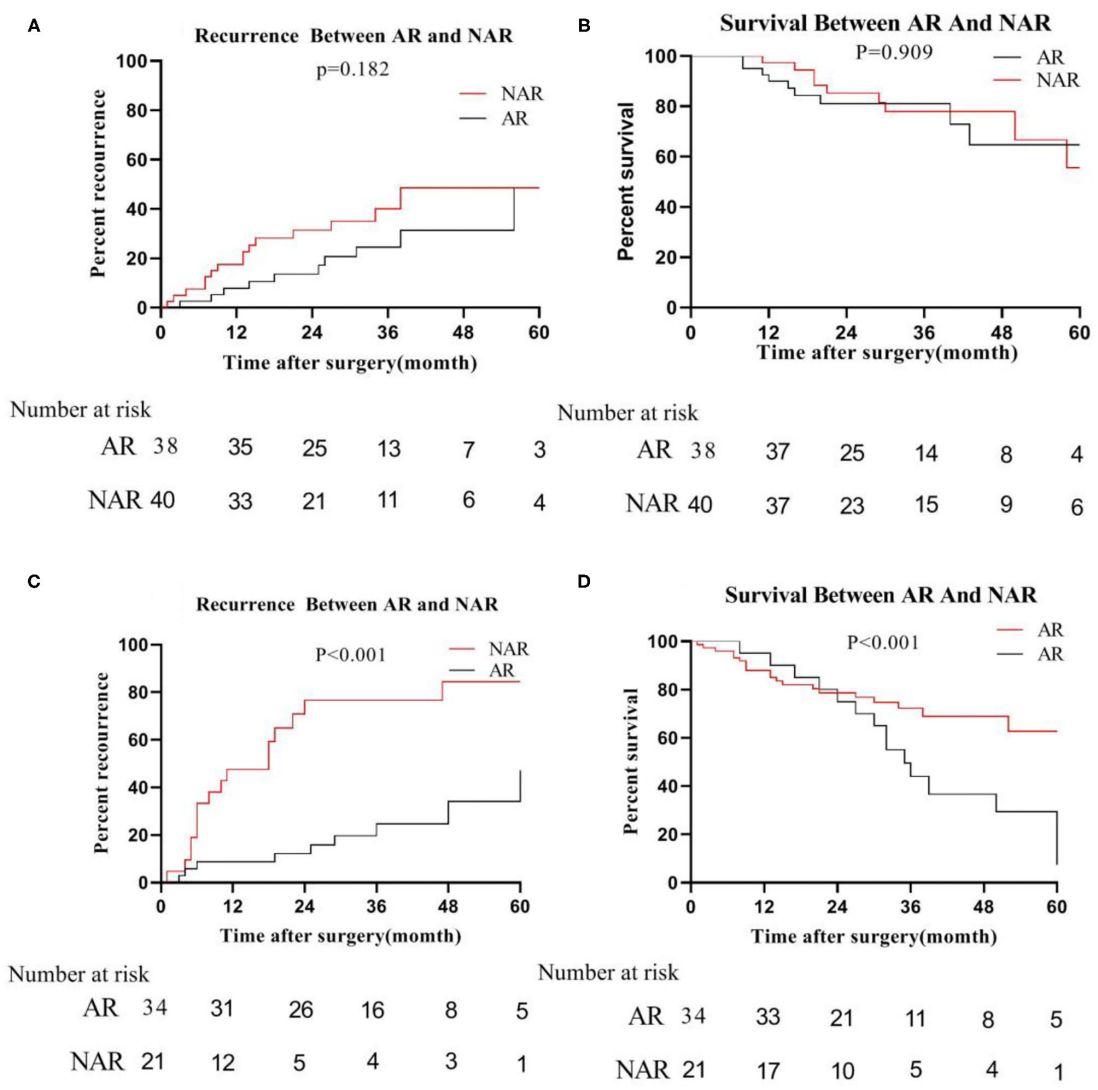
In the low-risk group, recurrence **(A)** and survival **(B)** between AR and NAR when tumor size ≦5 cm; recurrence **(C)** and survival **(D)** between AR and NAR when tumor size >5 cm.

However, in the low-risk group, we found that the median recurrence time of patients undergoing AR was 34 months for tumor size ≦5 cm, and 17 months in NAR liver resection. But, there was no significant difference between the two (*P* = 0.182; Figure 5A). The median survival time of patients with anatomical hepatectomy and non-anatomical hepatectomy was 36 and 24 months, respectively. Also, there was no significant difference from each other (*P* = 0.909; Figure 5B). For patients with tumors size >5 cm, the 5-year recurrence rate of patients undergoing anatomical liver resection was 48.0%, which was significantly lower than 82.8% of patients in NAR (*P* < 0.001; Figure 5C). Similarly, the 5-year survival rate of the AR group was also higher than that of the NAR group (73.0 vs. 14.2%; *P* < 0.001; Figure 5D).

## Discussion

In this work, a predictive model for HCC MVI is established by combining hematological indicators with imaging characteristics. The model includes alpha-fetoprotein, neutrophil-lymphocyte ratio, corona enhancement, and peritumoral hypointensity on HBP images, which is convenient and accurate. Anatomical liver resection is beneficial to the long-term survival of patients with high-risk of MVI. While in the lower risk group, anatomical liver resection for patients with tumors >5 cm in size will be more conducive to long-term survival. For those with a size ≦5 cm, both methods are acceptable.

MVI and AFP have been proved as independent risk factors of early recurrence and poor overall survival after liver cancer liver resection; the correlation between them has been dramatically focused. Furthermore, it also has attracted much scholars' attention to the epidemiological and molecular biological relationship between tumors and inflammation (24–26). The NLR reflects the antagonism of the body against tumors by reflecting the relative changes in neutrophil and lymphocyte counts. A recent study found that NLR >3.0–3.2 is an independent risk factor for MVI (8). Analogously, there was meta-analysis finding that NLR does have a significant correlation with vascular invasion. The analysis of 17 research also found that their NLR cutoff value range is from 1.51 to 5.0 (27). In our study, the cutoff value of NLR is 3.8, which is consistent with those researches.

Gadoxetic acid-enhanced MRI scanning could increase the detection rate of micro hepatocellular carcinoma significantly; besides, some studies have discovered that specific characteristics of MRI could be used as typical features of imaging diagnostic MVI, such as incomplete imaging capsule, coronal enhancement in arterial phase, peritumoral hypointensity on HBP images. Lee et al. (28) found that arterial peritumoral enhancement, non-smooth tumor margin, peritumoral hypointensity on HBP were characteristic risk factors that are indicating microvascular invasion of HCC. In this study, significant correlations were found between peritumoral hypointensity on HBP, corona enhancement, and MVI, and this model was included for this purpose.

Early prediction of MVI risk can benefit preoperative individualized treatment plans, which is a consensus among scholars. Xu et al. (13) constructed a predictive MVI model extracted from CT images using Radiomics technology, which obtained satisfactory prediction results (AUC = 0.909). However, the process is complicated since the feature extraction of Radiomics requires algorithms to be developed by science and engineering technicians. It is currently hard to be acknowledged and being put into clinical practice owing to the over-fitting or under-fitting of many algorithms to imaging. Many studies are focusing on the prediction of MVI based on preoperative hematological indicators, but the prediction effect is poor (0.744–0.774) (29). Our prediction model, which integrates hematological indicators with radiology imaging features is concise and operable. Furthermore, it has a higher prediction accuracy than Radiomics and single hematology index prediction models. The AUROC in the test cohort is 0.887, and the AUROC in the verification cohort is 0.938.

The presence of MVI will result in increased early recurrence rates and reduced long-term survival. In this study, all patients were divided into a higher-risk group and a lower-risk group by the Yoden index. The 3- and 5-year survival of the higher-risk group were lower than those of the lower-risk group (56.09 vs. 71.59%, 32.01 vs. 54.47%; *p* = 0.019). Our study finds that performing anatomical liver resection in high-risk groups of MVI is beneficial to patients' long-term survival. When Professor Makuuchi determined the definition of anatomical hepatectomy, he believed that a gross resection of the tumor-bearing liver removes not only the tumor visible to the naked eye but also microvascular invasion that is difficult to detect (30), which has been affirmed by many studies and is also consistent with the findings of our study. Also, some researchers revealed that AR or NAR for HCC with MVI did not influence the recurrence-free survival or OS rates after hepatectomy in the modern era (16).

However, our study discovered that even in the MVI low-risk group, patients with HCC could obtain long-time survival when performing AR. In order to eliminate the effect of different tumor diameters on recurrence and survival after liver resection, we performed a further analysis using the tumor diameter of 5 cm as the cutoff value according to the literature. In the low-risk group, whether patients undergoing anatomical liver resection did not affect their tumor-free survival rate and survival rate. However, performing anatomical liver resection for patients with tumor diameters >5 cm is beneficial to long-term survival. The probable reason is that larger tumor size is associated with capsular invasion, satellite nodules, tumor thrombi, and non-invasive growth patterns (31). Moreover, larger HCC tumor size stimulates invasive behavior.

Also, several shortcomings existed in this study. A single-center retrospective study and the number of data enrolled is insufficient, which might lead to some bias of the data. Dates were not further divided by tumor sizes during the enrollment process, which would also have an impact on the patient's prognosis. Some studies found that different tumor sizes (31, 32) and the shortest distance from the edge of the tumor to the plane of surgical margin (33–35) would significantly affect post-operative outcomes, yet no further discussion was done in the survival analysis in this study. The issues mentioned above need to be analyzed by further multi-center and extensive sample data. This work is ongoing in our center.

In summary, we have developed and validated a novel score for predicting MVI risk in patients with HCC. Due to a high risk of early tumor recurrence, our findings suggest that patients with high MVI risk should undergo AR rather than NAR at the time of initial treatment allocation. Furthermore, in patients with lower MVI risk when tumor size >5 cm executing AR is of great necessity, also.

## Data Availability Statement

The raw data supporting the conclusions of this article will be made available by the authors, without undue reservation.

## Ethics Statement

The studies involving human participants were reviewed and approved by Institutional Ethics Review Board of Zhujiang Hospital. Written informed consent for participation was not required for this study in accordance with the national legislation and the institutional requirements.

## Author Contributions

CF contributed to the conception of the study. HH performed the study. HH, SQ, SZ, and PZ contributed significantly to analysis and manuscript preparation. HH, SQ, LH, and SW performed the data analyses and wrote the manuscript. HH, NZ, JY, WZhang, WZhu, and NX helped perform the analysis with constructive discussions. All authors contributed to the article and approved the submitted version.

## Conflict of Interest

The authors declare that the research was conducted in the absence of any commercial or financial relationships that could be construed as a potential conflict of interest.
