# Supplementary material for: Importance of Microvascular Invasion Risk and Tumor Size on Recurrence and Survival of Hepatocellular Carcinoma After Anatomical Resection and Non-anatomical Resection
Source: Front Oncol. 2021 Mar 17;11:621622. doi: 10.3389/fonc.2021.621622 (PMC8010691; doi:10.3389/fonc.2021.621622)
Supplement: Supplementary file 1 [file Data_Sheet_1.PDF]

Supporting Information

Importance of microvascular invasion risk and tumor size on recurrence and survival of hepatocellular carcinoma after anatomical resection and non-anatomical resection

Haoyu Hu[a], Shuo Qi[a], Silue Zeng[a], Peng Zhang[a], Linyun He[a], Sai Wen[a], Ning Zeng[a, b], Jian Yang[a, b], Weiqi Zhang[a] ,Wen Zhu[a, b], Nan Xiang[a, b] Chihua Fang[a, b]

[a]Department of Hepatobiliary Surgery, Zhujiang Hospital, Southern Medical University, Guangzhou 510280, China

[b]Guangdong Provincial Clinical and Engineering Center of Digital Medicine, Guangzhou 510280, China

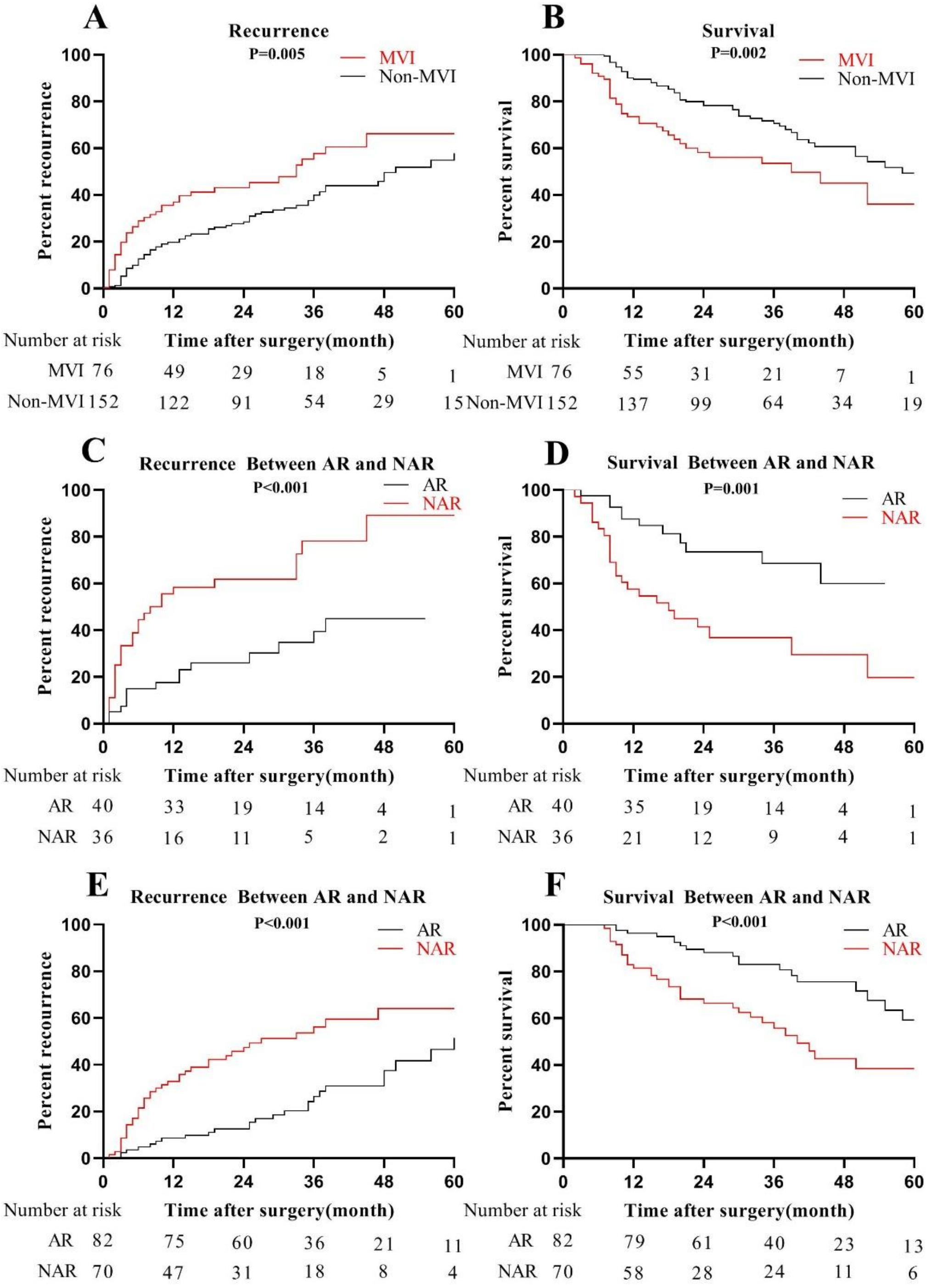

Figure.1 recurrence-rate(A) and survival-rate (B)comparison between MVI and Non-MVI group; In MVI group, recurrence-rate(C) survival -rate(D) comparison between AR and NAR; In Non-MVI, recurrence-rate(E) and survival -rate(F) comparison between AR and NAR.

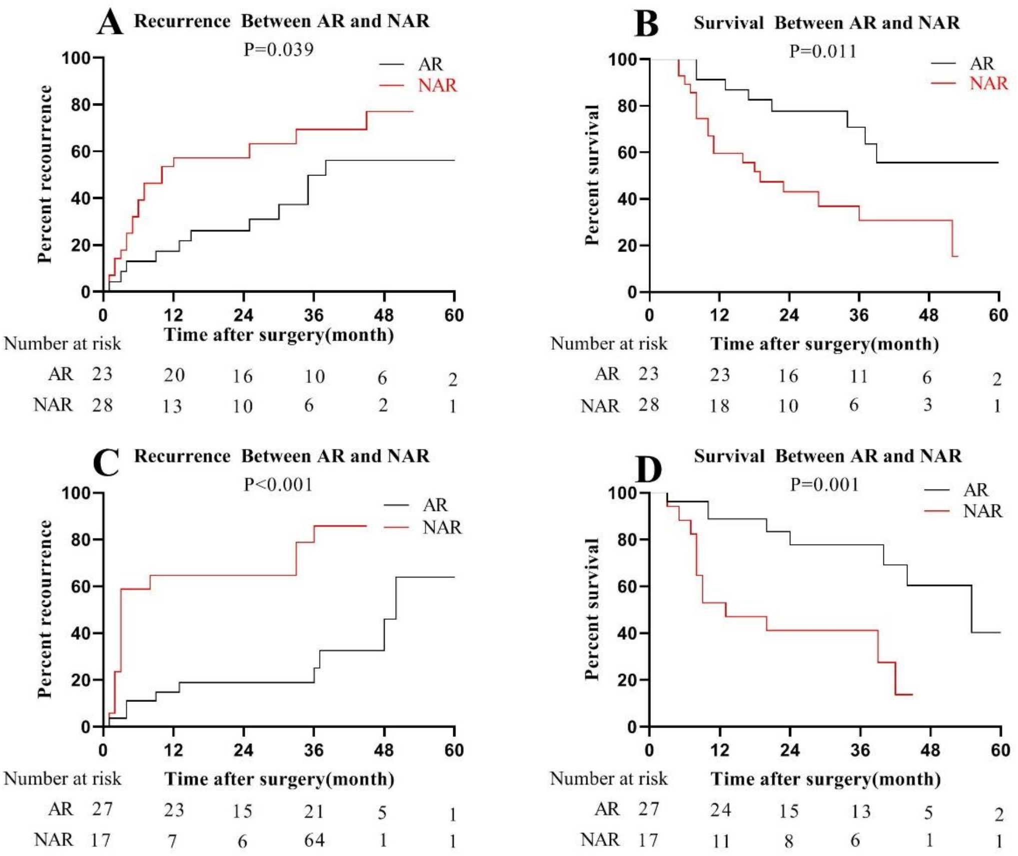

Figure.2 In the high-risk group, Recurrence(A) and Survival(B) between AR and NAR when tumor size≦5cm; Recurrence(C) and Survival(D) between AR and NAR when tumor size>5cm.
